# Analysis of internal processes of conflict behavior among Iranian rangeland exploiters: Application of environmental psychology

**DOI:** 10.3389/fpsyg.2022.957760

**Published:** 2022-08-31

**Authors:** Latif Haji, Dariush Hayati

**Affiliations:** Department of Agricultural Extension and Education, School of Agriculture, Shiraz University, Shiraz, Iran

**Keywords:** conflict behavior, rangeland degradation, norm activation theory, conflict management, environmental psychology

## Abstract

Conflicts over rangeland exploitation have been a serious challenge in Iran, rooted in human behavior. Accordingly, this study aimed to provide a comprehensive theoretical framework in the field of analyzing conflict behavior among rangeland exploiters. This research is a descriptive-correlational and causal-relational study conducted using a cross-sectional survey. The statistical population of the study was rangeland exploiters in one of the northwest provinces of Iran (*N* = 66,867) of whom 384 people were selected as a sample and stratified random sampling method with proportional assignment. The research instrument was a questionnaire, the validity of which was confirmed by a panel of academic experts and the reliability of its items was verified using Cronbach’s alpha coefficient. The results showed that the variables of personal norms (PN) and the perceived behavioral control were able to predict 25.9% of the variance in terms of the conflicting behavior of rangeland exploiters; besides, ascription of responsibility, PN, perceived behavioral control, and awareness of consequences, which have been proposed as activators of PN, were able to explain a significant percentage (63.5%) of the variance in terms of PN. Furthermore, analysis of the effects of environmental and cultural values showed that conflict behaviors of exploiters were mostly affected by their underlying values. Generally, the results of this study would help in the development of more integrated and comprehensive models in the field of exploiters’ conflict behavior. Eventually, to change and improve the environmental behavior of exploiters to better management of conflict in rangelands, providing a list of considerations and competencies for agricultural extension and education, this article comes to the end.

## Introduction

Rangeland degradation has turned into one of the most serious environmental issues in the world (Roudgarmi, 2013). Considering the special environmental and population status of Iran, this country, with 90 million hectares of rangeland (Iranian Ministry of Agriculture Jihad, 2019), has not been secured from such degradations over time (Azadi et al., 2009). It has been estimated that over the last three decades, more than 20% of its rangeland has been degraded in terms of quality and quantity (FAO, 2013; Haghiyan et al., 2016); this amount, occurred with greater intensity, is compared to the European and American countries (Haghiyan et al., 2016). A combination of factors, most related to human activities, is usually considered as the cause for this degradation (Harris, 2010; Savari and Gharechaee, 2020). According to FAO statistics, about 30% of the degradations were related to natural changes and effects, whereas about 70% were related to management and human activities (FAO, 2013). Among human factors, the competition in rangeland exploitation might be pointed out (Ubwa, 2018). Throughout history, human societies have had often challenges over the right to exploit natural resources (Green, 2002); in the present era, disputes over the exploitation of resources have reached their peak (Al-Muqdadi, 2019). One of the abnormal behaviors is social impacts in the form of conflict between individuals (McCollum et al., 2010). On the one hand, paying attention to the standards and facilities that human needs for his wellbeing and life has caused the acceleration of the exploitation of rangelands (Hill and Mustafa, 2011); on the other hand, the failure of communities to establish appropriate structures (governance) and preventive strategies for the conflict prevention provides a suitable environment for these conflicts (Collins, 2019). So, the combination of these factors has led to a decrease in the quality and quantity of rangelands (Adeoye, 2017).

These problems are more indicative of the fact that rangeland is a common pool resource (CPR) and could be available to all (Hileman et al., 2016; Haji et al., 2021). CPR is resource that first, the exclusion of stakeholders would be costly in any way (physical/institutional), and second, the exploitation of the resources by one exploiter reduces others’ access to them (Alipour and Arefipour, 2020). Hardin (1968) mentions this theorem as a tragedy of commons; “individuals who are sharing a common resource attempt to act in their own benefit, believing that they might obtain worse results than when they act collaboratively” (Blanco et al., 2019). This complex situation of common resources, such as rangeland, would be a platform for competition and conflict between exploiters (Ochola et al., 2010; Apipalakul et al., 2015; Adeoye, 2017). Analysis of different definitions has shown that “conflict” is a social situation, where two or more actors try to have more access to one or more resources at the same time (Veisi et al., 2020). Conflict occurs when the parties to the conflict have incompatible interests, goals, and values and try to achieve those goals (Yang et al., 2013). Environmental conflicts are basically intra-group conflicts that arise over tangible resources, such as water or land (Opotow and Brook, 2003), and they are mostly led to inequalities and social tensions (Selemani, 2014). When environmental contradictions get through a destructive trend, individuals in one group might negate other groups and ignore the ethical considerations (Opotow and Brook, 2003).

Looking deeply into natural resource management literature suggests that in these areas, three main trends can be identified that include economic, technological, and behavioral trends (Bijani et al., 2017; Haji et al., 2020). Many scientists and scholars believe that the discussion about individual behavior is more important than other factors (Steg and Vlek, 2009; Urech et al., 2013; Sadeghi et al., 2017). Hence, to prevent the decrease in the rangeland resources, development and reinforcement of appropriate behaviors among exploiters (as the largest users) seem to be necessary (Yazdanpanah et al., 2014; Haji et al., 2020). Therefore, knowing the way of people thinking, how they perceive about rangelands, their tendency toward different measures in the conservation of rangeland resources, and solving problems and crises related to that, it would be necessary (Katuwal, 2012) that the first step in this direction comes about understanding their current behaviors (Yazdanpanah et al., 2014). In this regard, as a suitable tool for understanding individuals’ behavior, environmental psychology and theories in this field of science have a special place in research sources (literature) (Wauters et al., 2010; Bamberg, 2013; Onwezen et al., 2013).

## Theoretical background

In the field of environmental psychology, there are usually two main approaches to pro-environmental behavior of individuals which are referred to as the “rational approach” and “moral approach” (Valizadeh et al., 2016). Each approach has its own special advocates who seek justifications to validate different approaches and theories. Rational approach assumes that human behavior is a rational choice situation (Steg and Vlek, 2009); on the contrary, the presupposition of the moral approach considers human behaviors as a moral perspective (Stern, 2000). A situation of rational choice is one in which one’s actions have consequences for the others’ welfare (Valizadeh et al., 2018a); in other words, theories like the theory of planned behavior (TPB) and theory of reasoned action (TRA) that fall within the domain of rational approach theories ignore moral considerations; besides, these theories mostly focus on the egoistic values (EV) and individualistic values (IV) of individual behavior (Kaiser et al., 2005), while in the next generation theories in the field of environmental psychology, other values have been formed, which, in addition to EV, have also taken into consideration altruistic values (AV), biospheric values (BV), and collectivistic values (CV) (Pradhananga et al., 2017). Nevertheless, the TPB and TRA lack rigid theoretical foundations in the crystallization of these values in individual behavior (Valizadeh et al., 2018b). This is while, researchers, who assume self-interests as the most important motivator for environmental behavior, mostly have used rational choice models (Bamberg and Möser, 2007; Valizadeh et al., 2018a; Savari and Gharechaee, 2020).

On the contrary, theories such as norm activation theory (NAT) and value-belief-norm theory (VBN) that are part of the theories put forward in the ethical approach, other than considering the AV and BV, take into account the IV either (Harland et al., 2007), but one of the major weaknesses in the theories of this approach is that it does not consider the impact of social relationships on behavior (Valizadeh et al., 2016). These interactions are especially evident in social challenges, such as conflict behaviors over CPRs as to rangelands, because there are many contradictions between “individual and collective” and “short-term and long-term” desires in the real world. This conflict (acting for an individual or collective interests) is generally manifested in the form of two cultural values, which are IV and CV (Valizadeh et al., 2021). Although environmentalist values show the different dimensions of human–environmental interactions, they do not consider human–human interactions (Valizadeh et al., 2016). Despite all available interpretations, the theories of this approach are widely applied in the sphere of environmental psychology; inasmuch as, in these theories, the relationships between the independent variables and the dependent variables of behavior are explained more clearly. Generally, ethical theories are more applicable in the context of various environmental behaviors and in the area of individual and collective behaviors (Kaiser et al., 2005; Harland et al., 2007; Nigbur et al., 2010).

**FIGURE 1:**
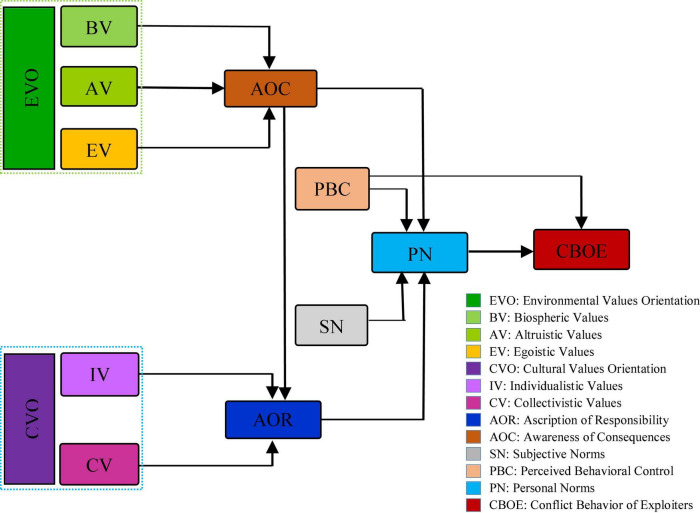
Conceptual framework of the study.

As stated in the theoretical background of the research, to overcome the shortcomings of each of the proposed theories and to increase their prediction power, the present research framework (Figure 1) is a developed form of three theories of norm activation (Schwartz, 1977), theory of VBN (Stern, 2000), and TPB (Ajzen, 1991). But, due to the fact that the main dependent variable in this study was the conflict behavior of individuals in rangeland exploitation and the nature of such behaviors was more matched with the goals and nature of the NAT, it was attempted to consider this theory as a basis for conceptualizing the research framework, because the issue of conflict in rangeland exploitation cannot be assessed in ecological analysis without considering the relationship between human and environment (which is a moral relationship) (Veisi et al., 2020). On the contrary, the moral norm is the core of NAT and the outcome of further conflict behaviors affects the morality of the exploiting community and fellowman (Bijani and Hayati, 2011). In this regard, some of the most important determinants, proposed in various studies as key predictors, were added to the theory of norm activation.

An important presumption of NAT is that personal (moral) norms (PN) are the main predictors of pro-environmental and pro-social (altruistic) behaviors (Klöckner, 2013; Onwezen et al., 2013; Shin et al., 2018). According to the NAT, the variable of PN is considered an immediate variable that affects the conflict behavior of exploiters (CBOE). Schwartz (1977) defines PN as the strong sense of moral commitment that drives individuals to participate in pro-social behaviors (Bamberg and Möser, 2007). According to Schwartz (1977), PN might be activated or deactivated by two belief constructs. These two structures include ascription of responsibility (AOR) and awareness of consequences (AOC) (Abrahamse and Steg, 2013; Lind et al., 2015); in other words, if a person is aware of problems arising from specific behaviors, this awareness would be then followed by his/her own contribution to those problems and the question that whether one can help in solving such problems or not (Abrahamse and Steg, 2013; Lind et al., 2015). In general, if one is aware that his/her behaviors have negative effects on others and also the environment (e.g., AOC), he or she could then feel responsible for those negative effects; so it could be believed that his/her responsible environmental behavior helps the reduction of the environmental problems (e.g., AOR) and consequently activates one’s PN (Eriksson et al., 2008; Steg and De Groot, 2010). In other words, an individual’s awareness of the problem being the first step toward responsible action, and the degree to which he is aware of solving problems through individuals’ behavior, in turn, activates one’s personal norm (Davis et al., 2015; Liu et al., 2017). Thus, the above explanations would lead to the following hypotheses:

**H1:** CBOE will negatively and significantly be affected by PN;

**H2:** PN will positively and significantly be affected by AOC;

**H3:** PN will positively and significantly be affected by AOR;

**H4:** AOR will positively and significantly be affected by AOC.

Other than AOC and AOR, there are other variables that have been considered in various studies as preconditions for PN. In this regard, Bamberg and Möser (2007) have argued that individuals’ ability to perform a behavior under their perceived behavioral control (PBC) is effective in the formation of PN. Also, other studies show that PBC can influence CBOE (Pradhananga et al., 2015, 2017; Valizadeh et al., 2021). PBC refers to an individual’s ability to successfully perform a behavior (Borges et al., 2014). Based on the NAT, if individuals feel that they have the ability to mitigate the ill effects of a behavior and have access to the resources and potentialities in this regard, then they would show a high level of personal commitment (Schwartz, 1977; Pradhananga et al., 2015). Subjective norms (SN) is defined as a person’s understanding of “what others care about?” (White et al., 2009) and “understanding social pressure to the perpetration or not perpetration of a behavior” (Ajzen, 1991). Probably, if individuals understand the importance of the confirmation of that behavior by others, then they would certainly show more commitment to do that (Ajzen, 1991; Wauters et al., 2010). There has been a lot of empirical support in various studies for the predictive effects of PBC and SN variables on the PN variable (Bamberg and Möser, 2007; Klöckner, 2013; Yazdanpanah et al., 2014; Pradhananga et al., 2017). Based on these arguments, the following hypotheses are proposed:

**H5:** CBOE will negatively and significantly be affected by PBC;

**H6:** PN will positively and significantly be affected by PBC;

**H7:** PN will positively and significantly be affected by SN.

There is much evidence for the inclusion of value orientation variables in the NAT. Values act as information filters that enable individuals to selectively accept or follow information (Sarrica et al., 2016; Pradhananga et al., 2017; Valizadeh et al., 2021). According to moral theories, PN are activated by the cognitive structure of individual values and beliefs (Schwartz, 1977). In fact, individuals act in a manner that is correspondent with their values (Klöckner, 2013; Sarrica et al., 2016); besides, it seems that various conflicts and disagreements over resources, such as rangelands, are mainly more related to the conflict of values than to rangeland’s resources (Valizadeh et al., 2021). To understand the environmental values of individuals, different frameworks and models have been used by researchers (Schwartz, 1977; Stern, 2000; Corral-Verdugo et al., 2006; Valizadeh et al., 2016), but the commonality of all is the “value diversity” that exists among different individuals in a community for resource valuation, such as rangeland (Valizadeh et al., 2021). These value orientations include environmental values (AV, BV, and EV) and cultural values (IV and CV) (Pradhananga et al., 2017).

Many scholars have confirmed the existence of a relationship between values, AOR, and AOC (Gärling et al., 2003; De Groot and Steg, 2009; Bijani and Hayati, 2013; Ives and Kendal, 2014; Pradhananga et al., 2017; Valizadeh et al., 2018a,^2021^). Accordingly, in this study, there has been an attempt to indirectly relate environmental and cultural values to the conflicting behavior of rangeland exploiters through the AOR and AOC variables (Figure 1). Based on this, the following hypotheses are proposed:

**H8:** AOC will positively and significantly be affected by BV;

**H9:** AOC will positively and significantly be affected by AV;

**H10:** AOC will negatively and significantly be affected by EV;

**H11:** AOR will negatively and significantly be affected by IV;

**H12:** AOR will positively and significantly be affected by CV.

According to the literature, it became clear that although there have been limited studies related to conflict behavior in Iran (Bijani and Hayati, 2013; Mohammadinezhad and Ahmadvand, 2020; Veisi et al., 2020), most of them have been in the field of water resources. Therefore, to the best of our knowledge, no comprehensive research has been conducted on conflict behavior in rangeland exploitation. Also, this study has tried to provide an integrated model of conflict behavior for its better management.

## Research methodology

### Research design

This study is an applied research in terms of its objective. This is because the results and recommendations of this research might be used by various stakeholders, such as managers of natural resources, watershed managers, managers of agricultural organizations, ranchers, and farmers. In addition, this study is a survey and cross-sectional study in terms of data collection, and time and quantitative research in terms of its nature, which follows the positivism paradigm. It is descriptive and causal-relational in terms of data and information analysis methods. Also, it is a field study in terms of monitoring and controlling variables to examine all variables in natural conditions.

### Study area

**FIGURE 2:**
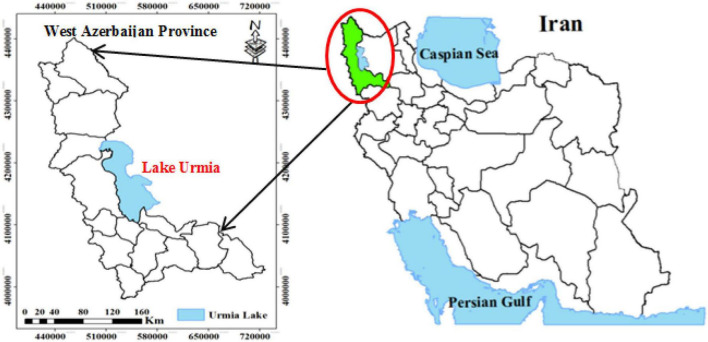
Site of the study area.

This research was carried out in West Azerbaijan Province, located in the northwest of Iran (Figure 2). Regarding the rural economy in West Azerbaijan Province, it should be noted that the rural economy of this province is mostly based on agriculture and animal husbandry. Rangelands constitute 60% of West Azerbaijan province, which play an important role in the economy and livelihood of rural households and exploiters. However, only 21% of the rangelands in the province are rich and have a high density. Statistics show that due to natural and human factors, 181,000 ha of the province’s natural areas have been turned into desert. Natural factors include climate change, reduced rainfall, and droughts, and the human factors include unprincipled exploitation, mining, local conflicts, rangeland plowing, bushes’ elimination, livestock increase, and excessive and unprincipled grazing, such as early spring grazing and non-compliance with the capacity and time of livestock arrival (West Azarbaijan Agricultural Jihad Organization [WAAJO], 2019).

### Statistical population and sampling method

The statistical population of the study consisted of all rangeland exploiters in West Azerbaijan Province, Iran (*N* = 115,066). Then, the studied area was divided into three parts, northern, central, and southern, and two counties were selected from each part (*N* = 66,867). Due to the fact that these counties were different in terms of the number of exploiters, the main sample size was divided between them in proportion to the volume. Using Krejcie and Morgan sampling table (Krejcie and Morgan, 1970) and a stratified random sampling approach, 384 exploiters were selected as the study sample. For sampling, a stratified random sampling method with proportional assignment was used (Table 1). The sample included respondents with a wide variety of social and demographic backgrounds. Also, all participants in the process of data collection were volunteers.

**TABLE 1:** West Azerbaijan rangeland exploiters and selected samples.

Parts	County	Population size	Sample size
North	Maku	8,500	49
	Khoy	14,755	85
Central	Urmia	19,424	111
	Naghadeh	3,905	22
South	Piranshahr	16,318	94
	Bukan	3,965	23
	Total	66,867	384

Statistical Annual of West Azer baijan Province [SAWAP] (2018).

### Survey instrument

A survey study was used to investigate and analyze the behavior of exploiters’ conflict in the use of rangelands. The research instrument was a researcher-made questionnaire (inspired by other researchers), whose validity was confirmed by a panel of experts. Cronbach’s alpha coefficients were used to determine the reliability. For this purpose, a pilot study, which included 30 ranchers, was conducted in an area outside the study area. Table 2 shows the research variables and the values of Cronbach’s alpha coefficients for each of them. Based on this coefficient, the reliability of the research instrument was between acceptable and good (0.74 ≤ α ≤ 0.89). After the pilot study and making the necessary revisions to the research tool, a questionnaire was prepared for the main survey phase. The questionnaire consisted of three parts, the first part was allocated to introducing the research title and objective, and the second part was related to the demographic characteristics of the respondents. Finally, the third section was related to the main variables in the theoretical framework (Figure 1) and the items for measuring each variable (Table 2). To assess and score the dependent variable (conflict behavior), a five-point Likert scale from 1 (never) to 5 (always) was used. Also, a five-point Likert scale from 1 (strongly disagree) to 5 (strongly agree) was used to assess and score the independent variables.

**TABLE 2:** Survey items and Cronbach’s alpha coefficients.

Variables	Items	Source
**Conflict behavior of exploiters**	**CBOE: conflict behavior of exploiters (α = 0.81)**	
	1. I do everything to get my share of the rangeland.	Veisi et al., 2020
	2. I have differences with government officials regarding how to manage the rangeland.	Veisi et al., 2020
	3. If possible, I will not allow other exploiters to access rangelands’ resources.	Bijani and Hayati, 2013
	4. If I do not get my share of rangeland exploitation, even if I am fined or imprisoned, I oppose the rangelands’ management and conservation.	Veisi et al., 2020
	5. If the natural resources department does not solve my problem, I will solve it personally.	Veisi et al., 2020
	6. I do not cooperate with community members to conserve rangeland resources.	Pradhananga et al., 2017
	7. I follow the laws related to rangeland conservation.	Self-administered
**Personal norms**	**PN: personal norms (α = 0.86)**	
	1. My personal values encourage me to consider the rights of others when using rangeland.	Shin et al., 2018
	2. I feel morally committed to preserving rangelands, no matter what others do.	He and Zhan, 2018
	3. When I participate in rangelands conservation activities, I feel I am a better (good) person.	Yazdanpanah et al., 2014
	4. I am committed to doing anything that can help reduce the vulnerability of rangelands.	Valizadeh et al., 2016
	5. Due to my own values and principles, I feel obligated to behave in a manner compatible with the environment.	Onwezen et al., 2013
**Subjective norms**	**SN: subjective norms (α = 0.74)**	
	1. People around me (my surroundings) want me to give up my interests when using the rangeland.	Self-administered
	2. When I participate in rangeland conservation activities, people around me will approve of me.	Yazdanpanah et al., 2014
	3. People around me believe that participation in rangeland conservation is a good job.	Yazdanpanah et al., 2014
	4. Friends and acquaintances want me to do whatever I can do to prevent rangeland degradation.	Self-administered
**Perceived behavioral control**	**PBC: perceived behavioral control (α = 0.89)**	
	1. I can easily participate in rangeland conservation activities.	Yazdanpanah et al., 2014
	2. I have the resources, time, knowledge, opportunities, and skills for rangeland conservation.	Pradhananga et al., 2017
	3. I am sure, I can put aside my interests when using the rangelands.	Shin et al., 2018
	4. I have the ability to change the way I use rangelands to conserve it.	Pradhananga et al., 2017
**Awareness of consequences**	**AOC: awareness of consequences (α = 0.84)**	
	1. I know that disputes over the use of rangeland can make the environment worse.	Shin et al., 2018
	2. Lack of optimal use of rangeland resources has caused a large migration of ranchers.	Valizadeh et al., 2016
	3. Lack of conservation of rangeland has been faced serious problems for the exploiters’ livelihood.	Valizadeh et al., 2016
	4. The negative consequences of the lack of rangeland resources in the future will be more worrying than we think.	Onwezen et al., 2013
**Ascription of responsibility**	**AOR: ascription of responsibility (α = 0.86)**	
	1. The local government (i.e., county, town/district) is responsible for maintaining rangeland quality.	Pradhananga et al., 2017
	2. Everyone must take responsibility for the environmental problems caused by the use of rangelands.	Shin et al., 2018
	3. It is the duty of the exploiters to conserve the rangelands, and the government alone is not responsible for it.	Valizadeh et al., 2016
	4. The current problems related to rangeland management are due to the incompetence of managers and have nothing to do with us exploiters.	Valizadeh et al., 2016
**Environmental values**	**BV: biospheric values (α = 0.85)**	
	1. Rangeland resources do not belong only to ranchers and farmers (humans), but must be consumed by other creatures (animals) that live in the rangelands.	Bijani and Hayati, 2013
	2. Rangeland vegetation should be preserved and people should not use it.	Bijani and Hayati, 2013
	3. Environmental protection and development have priorities over its use.	Bijani and Hayati, 2013
	4. I preserve rangelands for their intrinsic value.	Pradhananga et al., 2017
	AV: altruistic values (α = 0.84)	
	1. We do not have the right to think about rangeland conservation in a situation where rangeland exploiters are in difficult economic conditions.	Bijani and Hayati, 2013
	2. Since human beings are the supreme creature, meeting their needs is a priority.	Bijani and Hayati, 2013
	3. To rangelands optimal use, it is better that the exploiters pursue their interests less.	Valizadeh et al., 2016
	4. I preserve rangelands for the welfare of human beings.	Pradhananga et al., 2017
	EV: egoistic values (α = 0.73)	
	1. The rangelands and their exploitation belong only to me and others have no right.	Bijani and Hayati, 2013
	2. In using rangelands, I do not pay attention to the needs of others.	Bijani and Hayati, 2013
	3. The rangeland must first meet my needs and then its benefits reach the rest.	Bijani and Hayati, 2013
	4. It is only to meet my personal needs that I think of protecting rangelands.	Pradhananga et al., 2017
**Cultural values**	**IV: individualistic values (α = 0.75)**	
	1. In the exploitation of rangelands, I only follow my own personal goals, even if these goals are in conflict with the overall goals of society.	Pradhananga et al., 2017
	2. I would like to use and exploit rangelands in a way different from others.	Pradhananga et al., 2017
	3. The use of rangelands is a personal action and in this regard, I do not need to interact and cooperate with others.	Pradhananga et al., 2017
	CV: collectivistic values (α = 0.86)	
	1. I consider myself a part of the society I live in.	Pradhananga et al., 2017
	2. I have good cooperation and collaboration with people in different fields.	Pradhananga et al., 2017
	3. In trying to solve environmental crises, I try to adapt to the norms accepted by society.	Pradhananga et al., 2017

### Data collection and analysis

To reflect the views of rangeland exploiters, the data used in this study were collected through a questionnaire in West Azerbaijan province over a period of time (September–October 2020). The face-to-face method was used to collect survey data. Since the studied area and community had people with different cultures, languages, and customs, an interview group was formed before collecting data. This group consisted of five individuals who were fully acquainted with the culture, language, and customs of the local people. Since most interviewees had minimal education, in rare cases, the group of interviewers translated the questions for them during the face-to-face survey. After the briefing session with the interviewers, the research data were collected. At the end of data collection, from a total of 384 distributed questionnaires, 10 were excluded due to inappropriate and inadequate data; eventually, 374 questionnaires were analyzed using SPSS_22_ software. The data analysis was carried out in two parts. In the first part, which included demographic information, descriptive statistics [frequency, percentage, mean, and standard deviation (SD)] were used. In the second part, inferential statistics (Pearson’s correlation and multiple regression analysis) were used to analyze the relationships between variables. Also, path analysis was used to analyze the correlation decomposition between the variables and examine the direct and indirect effects of the variables.

## Results and discussion

### Descriptive analysis

Analysis of descriptive data showed that respondents were aged 17–71 years, and their mean age was 42 years (SD = 12.32). In terms of gender, 21 respondents (5.6%) were female and 347 respondents (92.8%) were male. The findings of education level in this study showed that 38 (10.2%) of subjects had academic education. In addition, descriptive findings showed that respondents had a minimum and a maximum of 2 and 55 years of experience in animal husbandry and the average livestock experience of subjects was 17.07 years (SD = 9.83). The investigation of individuals’ dependence on livestock showed that 73.5% of them had livestock dependence and had no other source of income. Regarding family members, the findings showed that 64% of the respondents were five or more. About 57% of ranchers had light livestock. Approximately 88% of people have experienced some kind of conflict (superficial to deep) in the last three years. Meanwhile, about 14% stated that the quality of the rangelands they use is in a good condition (44% is bad). Approximately 67.5% of them had not attended any rangeland conservation training classes. Also, about 13% believed that the government has more competence to manage, control, monitor, conserve, and exploit rangeland resources. Examining the results of descriptive statistics clearly can pave the way for conflicting behaviors among exploiters.

To obtain a qualitative description of the conflict behavior variable and classify respondents in terms of conflict behavior in rangeland exploitation, the interval standard deviation from mean (ISDM) method was used. This method is one of the popular choices for qualitative description of research variables (Alipour Amir et al., 2021). In ISDM method, the scores obtained are divided into four levels as follows (Table 3). The results showed that the rate of conflict behavior of 19.8% of exploiters was low, 21.4% of them were moderate, 22.2% was high, and 36.6% was very high. The results of Table 2 state about 60% of people had obvious conflict behavior over rangelands exploitation.

**TABLE 3:** Classifying the extent of conflict behavior of exploiters.

A < mean − SD	A < 25.64	Low	19.8%
Mean − SD < B < mean	25.64 < B < 28.17	Moderate	21.4%
Mean < C < mean + SD	28.17 < B < 30.70	High	22.2%
Mean + SD < D	C > 30.70	Very high	36.6%

Based on the results of this study, there are significant conflicts among rangeland’s exploiters in Iran. One of the main reasons for such a condition could be rooted in the rangeland management. Iranian rangelands came under the ownership and management of the government after the law on nationalization of forests and rangelands in 1963. This led each of the exploiters somehow engage in competition for maximum use of the rangelands, causing their destruction. It is obvious that in these conditions, no one will be held responsible for the current situation. On the contrary, according to a survey, only a limited percentage believed in public management of rangelands. Therefore, it is clear that such conditions will affect individuals’ behavior, and the type and extent of rangeland exploitation.

### Assessment of the relationships among variables

Pearson’s correlation test was used to investigate the relationships between variables (Table 4). According to the NAT, in which PN are the only variable that is directly related to behavior, this variable was directly related to behavior in this study (Figure 1), and the results showed that this variable has a negative and significant correlation with the CBOE (*r* = −0.488; *p* < 0.01). Based on this finding, it can be concluded that although exploiters are in conflict with each other over the use of rangeland, nevertheless, their moral commitment to the environment and other human beings reduces the conflict behavior. This result is consistent with Schwartz’s presumption (Schwartz, 1977) (that PN are the main predictor of altruistic behaviors) and has also been supported by empirical studies (Harland et al., 2007; Pradhananga et al., 2015; Valizadeh et al., 2021). On the contrary, according to the stated theoretical presuppositions, the four variables of the AOR, SN, PBC, and AOC are considered as the main drivers of PN (sense of moral commitment). Analysis of correlation relations in this section showed that all four variables of the AOR (*r* = −0.578; *p* < 0.01), SN (*r* = −0.416; *p* < 0.01), PBC (*r* = −0.395; *p* < 0.01), and AOC (*r* = −0.517; *p* < 0.01) have a negative and significant correlation with the CBOE. Among these, the values of correlation coefficients of the variables of AOC and AOR were higher than the other two variables, respectively. This indicates that these two variables are probably more capable than the other variables of activating PN. This finding shows that awareness of the consequences of conflict among exploiters, as well as their responsibility in relation to society and the environment, can reduce individuals’ conflict behavior.

**TABLE 4:** Correlation matrix of the theoretical framework variables.

	CBOE	PN	SN	PBC	AOC	AOR	BV	AV	EV	IV	CV
CBOE	1										
PN	−0.488**	1									
SN	−0.416**	0.624**	1								
PBC	−0.395**	0.534**	0.728**	1							
AOC	−0.517**	0.748**	0.616**	0.638**	1						
AOR	−0.578**	0.728**	0.568**	0.558**	0.805**	1					
BV	−0.429**	0.474**	0.694**	0.804**	0.546**	0.557**	1				
AV	−0.426**	0.396**	0.637**	0.818**	0.513**	0.483**	0.821**	1			
EV	0.613**	−0.581**	−0.536**	−0.574**	−0.665**	−0.604**	−0.498**	−0.500**	1		
IV	0.641**	−0.727**	−0.595**	−0.587**	−0.731**	−0.669**	−0.518**	−0.490**	0.804**	1	
CV	−0.439**	0.775**	0.639**	0.672**	0.783**	0.771**	0.577**	0.488**	−0.665**	−0.719**	1

CBOE, conflict behavior of exploiters; PN, personal norms; SN, subjective norms; PBC, perceived behavioral control; AOC, awareness of consequences; AOR, ascription of responsibility; EV, egoistic values; BV, biospheric values; AV, altruistic values; IV, individualistic values; CV, collectivistic values.

**Significant level: 0.01 error.

VBN and NAT theories and the results of some empirical studies have emphasized the indirect effect of environmental values (BV, AV, and EV) and cultural values (IV and CV) on an individual’s behavior (Stern, 2000; Ives and Kendal, 2014; Pradhananga et al., 2017; Valizadeh et al., 2021). In this study, cultural values and environmental values were indirectly related to conflict behavior in the rangeland exploitation through the AOR and AOC variables. The findings from the correlation of dual cultural values with the AOR showed that variables of IV (*r* = −0.669; *p* < 0.01) and CV (*r* = 0.771; *p* < 0.01) are correlated with the AOR in the field of rangeland exploitation. As expected, BV (*r* = 0.546; *p* < 0.01) and AV (*r* = 0.513; *p* < 0.01) had a positive and significant correlation with the AOC in the field of rangeland exploitation, and the variable EV had a negative and significant relationship with AOC (*r* = −0.665; *p* < 0.01). The contradiction between EV and AOC can be argued from the aspect that economic motivation is more important in the exploitation of rangeland, and this portrays a kind of human selfishness. As a result, this issue has caused exploiters to have distanced themselves from the principles of environmental sustainability and do not pay attention to the consequences of rangeland resource’s lack. This result highlights a competitive feature in the face of rangeland resource scarcity.

### The analysis of causal relationships among variables

**FIGURE 3:**
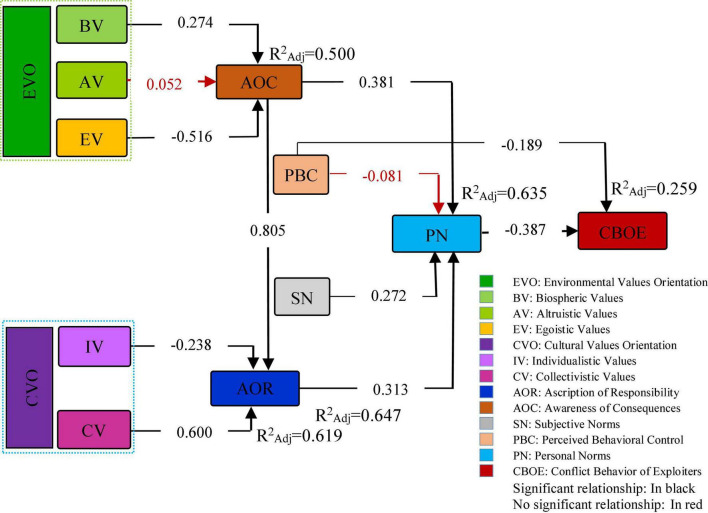
The causal research framework.

The results of multiple regression analysis showed that the causal model of the study was able to predict 25.9% of the variance in CBOE, 63.5% of the variance in PN, 61.9% of the variance in the AOR, and 50% of the variance in AOC (Table 5 and Figure 3). To facilitate the calculations related to path analysis, the theoretical framework of the research was first divided into four parts (sub-models), and in the next step, multiple regression analysis (ENTER) was used. In the first stage of causal analysis (the first sub-model), the direct effect of the variable of PN (moral commitment) and the PBC on CBOE was examined. The theoretical framework of this research is basically based on the NAT of Schwartz (1977), and in this theory, the PN (personal commitment) of the individual is considered one of the most important determinants of behavior. On the contrary, it is clear that the ability of individuals to control their behavior has a significant impact on the occurrence and non-occurrence of a behavior. Perceived ability to reduce risks refers to individuals’ beliefs about their ability to act and reduce the adverse effects of a behavior (Yazdanpanah et al., 2014). So that, if people have enough knowledge, skills, and information, they can better take steps to protect rangelands and use them optimally, thus reducing conflicts over the use of rangeland resources. Of course, according to the theoretical framework of the research, the results showed that the CBOE is more influenced by PN, which means that the variable of PN has a significant role in reducing conflict behavior among exploiters. As in the research framework evident, PN (personal commitment) is rooted in the variables of AOR, AOC, SN (directly), and cultural and environmental values (indirectly). The output of the results showed that PN (β = −0.387; *p* < 0.001) and PBC (β = −0.189; *p* < 0.001) in the field of rangeland exploitation have a negative and significant effect on the conflicting behavior of exploiters. This indicates that the more the sense of commitment and PBC of individuals in the exploitation of rangelands, the less the conflict behavior will be among them. This is supported by the findings of other researchers (Stern, 2000; Harland et al., 2007; Pradhananga et al., 2015).

**TABLE 5:** Calculation of direct effects on CBOE, PN, AOR, and AOC.

	Independent variables	*B*	Beta (β)	*T*	Significant *T*
Direct effects on the CBOE	Constant	35.289	–	53.626	0.001
	PBC	−0.248	−0.189	−3.58	0.001
	PN	−0.573	−0.387	−7.33	0.001
	Significant *F* = 0.001	*F* = 66.250	*R*^2^_Adj_ = 0.259	*R*^2^ = 0.263	*R* = 0.513
Direct effects on the PN	Constant	1.144	–	3.336	0001
	AOC	0.345	0.381	6.613	0.001
	PBC	−0.072	−0.081	−1.662	0.097
	SN	0.359	0.272	5.659	0.001
	AOR	0.297	0.313	5.858	0.001
	Significant *F* = 0.001	*F* = 163.010	*R*^2^_Adj_ = 0.635	*R*^2^ = 0.639	*R* = 0.799
Direct effects on the AOR	Constant	1.729	–	5.969	0001
	AOC	0.766	0.805	26.143	0.001
	Significant *F* = 0.001	*F* = 683.465	*R*^2^_Adj_ = 0.647	*R*^2^ = 0.648	*R* = 0.805
Direct effects on the AOC	Constant	16.425	–	15.377	0.001
	BV	0.353	0.247	3.800	0.001
	AV	0.073	0.052	0.792	0.429
	EV	−0.838	−0.516	−12.014	0.001
	Significant *F* = 0.001	*F* = 125.579	*R*^2^_Adj_ = 0.500	*R*^2^ = 0.505	*R* = 0.710
Direct effects on the AOR	Constant	8.106	–	6.863	0.001
	IV	−0.380	−0.238	−5.179	0.001
	CV	0.748	0.600	13.052	0.001
	Significant *F* = 0.001	*F* = 304.318	*R*^2^_Adj_ = 0.619	*R*^2^ = 0.621	*R* = 0.788

CBOE, conflict behavior of exploiters; EV, egoistic values; BV, biospheric values; AV, altruistic values; IV, individualistic values; CV, collectivistic values; AOR, ascription of responsibility; AOC, awareness of consequences; SN, subjective norms; PBC, perceived behavioral control; PN, personal norms.

In the second stage of path analysis (second sub-model), the effects of PN activators (AOR, SN, PBC, and AOC) on the moral commitment of exploiters were examined. The results of this section showed the three activators of the AOR (β = 0.313; *p* < 0.003), SN (β = 0.272; *p* < 0.001), and AOC (β = 0.381; *p* < 0.001) have positive and significant effects on PN, but PBC (β = −0.081; *p* < 0.097) had no significant effect on the PN. According to the findings of this stage, AOC had a positive relationship with PN and was one of the most important activators of PN. This indicates that providing information about adverse environmental consequences of rangelands and their impact on the quality of exploiters’ life is likely to reinforce pro-environmental norms (personal commitment). As the descriptive statistics show, most of the exploiters have low education and this leads to the impossibility of using the sources of information about the crises that affect rangelands. Also, the research results showed that the AOR has a positive effect on the exploiters’ PN. So that if people feel responsible for the rangelands, their moral commitment will increase and they will try to better preserve the rangelands; consequently, they show less conflict. One of the main reasons for the conflict and the destruction of rangelands is individuals’ irresponsibility. So in the conservation of rangelands, most exploiters consider the government as the only organization responsible for this. If they do not take responsibility for the existing problems of the rangelands and consider all the problems to be due to mismanagement of officials, the possibility of activating their moral commitment to play a role in reducing the adverse effects of the rangeland crisis will be reduced. Finally, regarding the effect of SN on PN among exploiters, it should be noted that social pressures in the form of SN can activate the moral commitment of individuals and thus reduce their conflicting behavior. The requirement of such SN also depends on the context or social environment of the exploiters and their attitudes and beliefs. The social environment through social pressure can reinforce the sense of moral commitment to preserve rangelands. Pradhananga et al. (2015, 2017), and Valizadeh et al. (2021) report similar results in their research and conclude that the three variables of AOR, SN, and AOC have positive and significant effects on the PN in the field of water conservation behavior. However, their results in terms of the effect of PBC on behavior are not consistent with the results of this study. The unique circumstances of the subjects and the nature of the study regarding the insignificance effect of PBC on PN can be justified. Therefore, this assumption should not be generalized to all communities. Sometimes, people have somehow moral commitment to the environment and communities; however, due to a set of factors (mostly profit motives), they cannot adhere to ethical issues (inability to control behavior).

In the third stage of path analysis, the effects of three environmental values (BV, AV, and EV) on AOC were examined and the findings showed that BV and EV significantly affected the AOC. Meanwhile, BV (β = 0.247; *p* < 0.001) positively and EV (β = −0.516; *p* < 0.001) negatively affected the AOC. The results of the previous studies (Pradhananga et al., 2017; Veisi et al., 2020; Valizadeh et al., 2021) support this finding, but the effect of AV on the AOC was not significant.

In the fourth stage, the effects of dual cultural values (IV and CV) on the AOR among exploiters were examined. The results of this stage of the causal analysis indicated that IV (β = −0.238; *p* < 0.001) had a negative and significant effect and CV (β = 0.600; *p* < 0.001) positively and significantly affected the AOR. Pradhananga et al. (2017) reported a similar result in their study.

In the third and fourth stages of path analysis, the variables of AOC and AOR were assumed as mediating variables between environmental values and cultural values with PN, respectively. The results of the causal relationship analysis showed that the two environmental values (BV and EV) had a significant contribution to explaining the AOC (0.50%). Also, cultural values (IV and CV) had a significant contribution to explaining the AOR (61.9%). This is important in these respects that two variables of AOC and AOR are the main determinants of the personal commitment of individuals for engagement in rangelands’ resource exploitation activities. It is obvious that individuals have diverse values and act accordingly. For example, individuals with CV, BV, and AV in rangeland conservation programs may focus on environmental and community benefits and emphasize the conservation of rangeland resources as a collective responsibility. In contrast, individuals with egoistic and IV, only if they believe their personal interests are threatened, feel a sense of personal commitment (Stern, 2000). Therefore, these people will better respond to programs that offer special benefits. Finally, the effects of AOC on AOR were examined. The results of this step showed that AOC (β = 0.805; *p* < 0.001) has a positive and significant effect on AOR.

### Correlation decomposition among research variables

To create a suitable context for understanding the relationships and causal mechanisms among the main variables of the research, the correlation values (*r*) and the standardized impact coefficients (β) were linked using correlation decomposition (Table 6). In this context, it should be noted that the PN variable had no indirect effect since it directly affected on CBOE. While the PBC variable had a direct effect on CBOE and its indirect effect was not significant. Also, environmental values (BV, AV, and EV) and cultural values (IV and CV), AOR, SN, and AOC were indirectly related to CBOE and had no direct effects. Calculation of indirect effects showed that the variables of AOC [(0.804 × 0.313) × (−0.387) + (0.381) × (−0.387) = −0.244], AOR [(0.313) × (−0.387) = −0.121], and SN [(0.272) × (−0.387) = −0.105] had the most indirect effects on CBOE. The total (causal) effects also indicated that the variables of PN (−0.387), AOC (−0.224), PBC (−0.158), AOR (−0.121), and SN (−0.105) had the highest causal effects, respectively. It can be concluded that based on the literature and framework provided in this study, although the variables of AOC, AOR, and SN did not directly affect the CBOE, their total effects indicate their key and determining role in the occurrence and explanation of the behavior. Therefore, strengthening these variables among exploiters can minimize the conflict over the use and competition of rangelands among them.

**TABLE 6:** Analysis of direct, indirect, and total effects of the variables on conflict behavior of exploiters.

No.	Variables	Direct effects	Indirect effects	Total effects
1	PN	–0.387	–	–0.387
2	SN	–	–0.105	–0.105
3	PCB	–0.189	0.031	–0.158
4	AOC	–	–0.244	–0.244
5	AOR	–	–0.121	–0.121
6	BV	–	–0.067	–0.067
7	AV	–	–0.012	–0.012
8	EV	–	0.026	0.026
9	IV	–	0.028	0.028
10	CV	–	–0.072	–0.072

## Conclusion and managerial recommendations

As mentioned, various stakeholders are involved in the use and exploitation of rangelands. On the contrary, it should be noted that rangelands are part of CPRs and their use has caused problems and issues for exploiters. Accordingly, this issue has created conflicts among exploiters as the main and most important stakeholders. Therefore, this research sought to determine what factors and processes affect the occurrence of conflict behavior by exploiters. So that identifying the causes and predispositions or backgrounds of conflict behavior can be useful in people’s understanding and thus trying to resolve conflict. One of the original contributions of this research was that in the field of conflict behavior in rangeland exploitation, special attention should be paid to behavioral and cognitive-social dimensions other than technical dimensions. For this purpose, in this study, the use of environmental psycho-social models that examine the relationship between humans and the environment can be useful and action guides for managers in this field.

Based on the framework provided in this study, which was based on the NAT and with contributions of VBN and the TPB, the research results showed that the structures of environmental psychology theories create a suitable framework for explaining conflict behavior in rangeland exploitation. In other words, the framework used in this study leads to the development and expansion of understanding in the field of complex interactions that exist between cognitive-behavioral variables of rangeland use. In the NAT, the sense of moral commitment of the individual had been raised as the most important and powerful variable in predicting behavior, which was the same in this study. The results showed that this variable alone could predict a significant share of variance changes in rangeland exploiters’ conflict behavior.

In addition, AOR, SN, and AOC variables were considered the main actuators for PN based on the rationale stated in the theoretical literature and they were able to predict the acceptable variance changes in terms of PN. On the contrary, the results of this study showed that to explain the conflict behavior in the rangelands’ exploitation (which is a kind of egoistic and individualistic behavior) and to make appropriate behavioral changes in reducing conflict and conserving rangelands, attention should be first paid to the value foundations (environmental and cultural) of the rural community and then to the exploiters’ moral commitment (PN), which is the strongest motivator and driver for their behavior. The results of this study provide practical implications for managers of forests, rangelands and watershed management organizations, environmental conservation managers, natural resources officers, intervention organizations, and even ranchers or exploiters themselves to be able to help achieve sustainability in the field of natural resources, especially rangelands. In addition, these findings can be considered by other countries (especially Iran’s neighbors) that have a similar situation to Iran, because the continuation of this situation can also affect the international situation. The importance of psychological and cognitive dimensions in the field of rangeland resource management and exploitation emphasizes the need to pay attention to these variables and determinants. In this regard, a list of considerations and competencies of agricultural extension and education to change and improve the environmental behavior of different stakeholders and thus manage the conflict among them (especially exploiters) is presented:

• Given the special cultural conditions of the community as well as the quality of rangelands in different regions, authorities are suggested to pay sufficient attention to private and participatory rangeland management. In this regard, the use of successful experiences toward local communities’ governance in natural resources management can be useful. Iran has different regional cultures. Thus, cultural and value differences affect people’s relationships with each other and with the environment. This increases the conflict over rangeland exploitation. On the contrary, rangeland management has been centrally owned by the government since 1963. Therefore, to better manage rangelands and consequently manage the conflict among exploiters, the management structure governing rangelands should be reformed and improved. Therefore, reduction of the transfer of responsibility and management of rangelands to the local level is suggested. For example, the establishment of organizations, such as rangeland cooperatives, can be very useful in the participatory management of local communities. Of course, applying this recommendation to other countries that have similar conditions to Iran can also be considered.
• Policymakers and managers, whose goal is to preserve and rehabilitate rangelands and their sustainability, should focus on those strategies that promote ethics and institutionalize it among the general public, especially the exploiters. Extension agents could be the best actors to achieve such a goal.
• Holding training classes in the form of capacity building, attracting attention to the rights of others, and the needs of the future generations can attract the attention of rangeland conservation authorities. Accordingly, the design of training programs and behavior change interventions should be such that they empower rangeland resource exploiters to overcome their behaviors. In this regard, they should be taught that conflict behavior can lead to the destruction of rangelands as much as possible.
• To strengthen the sense of responsibility among exploiters, steps can be taken to encourage civil liability to preserve rangelands by creating communication campaigns and NGOs. Also, the role of enlightenment agents of change at organizations in charge of rangeland conservation (especially the agricultural extension and natural resources organization in Iran) can be very useful, because they can explain the multiple dimensions (managerial, economic, and technical) to exploiters by implementing enlightenment programs and convince them that part of the current problems in the field of shortage and destruction of rangelands has been caused by humans. Thus, human beings themselves can help solve those problems by accepting responsibility in this field.
• The social environment through social pressure can strengthen the sense of moral commitment to preserve rangelands. Considering the undeniable role of SN and perceptions of those around that are related to the conflicting behavior of rangeland exploiters, local leaders, and community pioneers in different societies could play an effective role to overcome this problem.
• Programs and designs of rangeland resources’ conservation can be useful to increase the awareness of exploiters toward the consequences of environmental crises in rangelands and increase their responsibility. At first, they must reconstruct their underlying values. Engaging stakeholders in solving environmental problems (especially rangelands) can be useful to strengthen the values (environmental and cultural). Also, those in charge of rangeland affairs (in Iran, the Forests, Rangelands, and Watershed Management Organization, as well as public participation units) must understand the importance of diversity of values among exploiters and pay attention to the role of these values in their participation to conserve rangelands. This will increase the power of conflict management.

### Research limitations

It shall be noted that this study has limitations, and considering them could be useful for future studies. This study was quantitative research and was conducted based on a survey among the rangeland exploiters of West Azerbaijan Province in Iran. A second limitation was related to the cultural and linguistic differences of the study population. Although this issue increases the generalizability of the results, it should not be forgotten that it may cause problems in data collection. The instrument based on the translation of items constructed in other contexts may have lacked sensitivity in recognizing culturally relevant processes. Furthermore, the questionnaire administered by our research team, and given the particularly sensitive subject matter, may have introduced biases related to the interviewer–interviewees communicative relationship. So, we recommend that future researchers be sensitive to these issues in their studies. Therefore, the use of mixed methods (quantitative and qualitative) may provide more meaningful insights in future research. A third limitation was related to the type of variables. In this study, only the internal factors that affect conflict behavior were discussed from the perspective of psycho-social patterns and variables, not external factors. In this regard, future research should focus on the external causes of conflict in rangeland.

## Data availability statement

The raw data supporting the conclusions of this article will be made available by the authors, without undue reservation.

## Ethics statement

The studies involving human participants were reviewed and approved by the DH, Department of Agricultural Extension and Education, School of Agriculture, Shiraz University, Shiraz, Iran. Written informed consent to participate in this study was provided by the participants’ legal guardian/next of kin.

## Author contributions

Both authors were involved in all stages of the study, including theoretical studies, data collection, analysis and data processing, and the presentation of the report.
